# Limited evidence for graft selection in pediatric ACL reconstruction: a narrative review

**DOI:** 10.1186/s40634-022-00448-0

**Published:** 2022-01-14

**Authors:** Gianni De Petrillo, Thierry Pauyo, Corinna C. Franklin, Ross S. Chafetz, Marie-Lyne Nault, Louis-Nicolas Veilleux

**Affiliations:** 1 Shriners Children- Canada , 1003 Decarie Blvd, QC H4A 0A9 Montreal, Canada; 2 Department of Surgery, McGill University, Montreal, Canada; 3 Shriners Children- Philadelphia, Philadelphia, USA; 4 Ste-Justine University Health Center, Montreal, Canada; 5 Department of Surgery, Université de Montréal, Montreal, Canada

**Keywords:** ACL reconstruction, Individualized, Pediatric, Tear, Graft selection, Recovery, Return to sport

## Abstract

Anterior cruciate ligament reconstruction is the preferred treatment to anterior cruciate ligament injury. With the increase in anterior cruciate ligament injuries in both adults and skeletally immature patients comes the need for individualized anterior cruciate ligament reconstruction graft selection whether it is the type of graft (auto vs. allograft) or the harvesting site (hamstrings, iliotibial band, quadriceps, patella). Several factors need to be considered preoperatively in order to optimize the patients’ recovery and restore anterior cruciate ligament strength and function. These include age and bone maturity, preoperative knee flexor/extensor strength, sport participation, patient’s needs and anatomical characteristics. This paper aims at bringing evidence supporting the use of a personalized approach in graft selection for faster and more efficient return to sport and propose a theoretical framework to support the approach.

## Introduction

In minors aging between 3 and 20 years old, it is estimated that 50 per 100 000 population undergo anterior cruciate ligament reconstruction (ACLR) [1]. A significant increase in youth sports participation, year-round training, and competition, as well as early sport specialization has contributed to a greater incidence of anterior cruciate ligament (ACL) tears in skeletally immature athletes [2, 3]. If left untreated, ACL-deficient knees can result in early degenerative cartilage wear, chronic effusion, functional instability, meniscus tears, and subsequent osteoarthritis and lower rates of return to pre-injury level of sport [4, 5]. Therefore, ACLR is the preferred treatment for ACL tears with the end goals being to restore function, return to sport (RTS) and prevent further injuries [6]. The current literature has shown significantly higher graft failure rates in pediatric patients compared to their adult counterparts [7–9]. In fact, young age itself has been reported as a predictor of revision surgery regardless of patient sex [7–9]. Many factors have been suggested to play a role in the higher failure rates observed in young patients, such as higher activity levels, lower compliance to rehabilitation protocols, and higher anxiety levels [7, 8]. Several factors need to be considered when planning and performing ACLR to increase the likelihood of patients returning to pre-injury strength and function levels, such as the patients’ goals, needs, activity level, age, sex, pre-existing literature, and a surgeon’s preference.

The aim of this review is to generate discussions regarding the factors that should be considered for graft selection for ACLR in pediatric patients and to identify which aspects of pediatric ACLR require more scientific evidence. These include allografts, quadriceps tendon (QT) autografts, hamstrings tendon (HT) autografts, patellar tendon (BPTB) autografts, and iliotibial band (ITB) autografts. Due to the paucity of studies investigating the comparisons between different graft types for ACLR specifically in patients below 18 years of age, this article also reviews data from studies in which pediatric patients make up a fraction of the sample group.

### Patient-related factors driving graft selection

#### Sport participation

Considering that there is no perfect graft for all patients undergoing ACLR, surgeons should consider a multitude of factors before performing their surgery such as the patient’s sports participation. Soccer players, sprinters, and judo athletes may want to avoid the possibility of a postoperative knee flexor deficit which is associated with the use of a hamstring autograft for ACLR [10–12]. Knee extensors play a predominant role in the ground phase of running and are crucial to turns and tackles during soccer [13, 14]. Their role in joint stability increases with limb velocity during the practice of the sport [13]. Interestingly, differences in knee extensor strength have also been found to differ between levels of soccer players with elite athletes having the highest strength and amateur athletes the lowest strength [13]. Other athletes, such as volleyball players or alpine skiers mainly utilize knee extensors for bilateral jumping and situation-dependant knee joint loading, respectively [15, 16]. These athletes may want to avoid postoperative knee extensor deficits, which have been associated with the use of a quadriceps or a patellar autograft for ACLR [10–12, 17–19].

#### Sex

Young female patients are predisposed to a natural imbalance of quadriceps to hamstring activation during activity [20]. Therefore, the use of a HT autograft in this patient population may exacerbate this imbalance and increase the risk of re-injury [20]. Quadriceps to hamstrings strength ratios should be measured preoperatively. Then, prehabilitation exercise programs can be used to address abnormal preoperative quadriceps to hamstrings ratios. This may avoid short- and long-term postoperative complications and re-injury. Lesevic et al. (2020) compared knee extensor and flexor strength between men (*n* = 79) and women (*n* = 87) who underwent isolated ACLR with either BPTB or HT autografts [21]. They found that female patients receiving a HT autograft had a significantly lower mass-normalized knee flexor peak torque than females and males receiving BPTB autografts and males receiving HT autografts 6 months postoperatively [21]. Knee flexor weakness, as measured in this patient group, can accentuate knee muscle imbalances [21]. Therefore, female patients receiving HT autografts for ACLR may be at a higher risk of reinjury within the 6 months following ACLR [21]. Despite the evaluations being at 6 months post-op in this study, patients undergoing ACLR should follow individualized exercise prescriptions postoperatively because strength deficits can persist up to 2 years after ACLR [21]. Based on these findings, rehabilitation protocols may need to emphasize hamstring strengthening in female patients receiving HT autografts for ACLR [21]. Another sex difference exists regarding ACLR. Female patients’ tendons tend to be smaller in diameter than male patients’ tendons. As imbalances of quadriceps to hamstring activation and small tendon diameters are not exclusive to females, their assessment should be completed for all patients undergoing ACLR.

#### Graft diameter

**Fig. 1:**
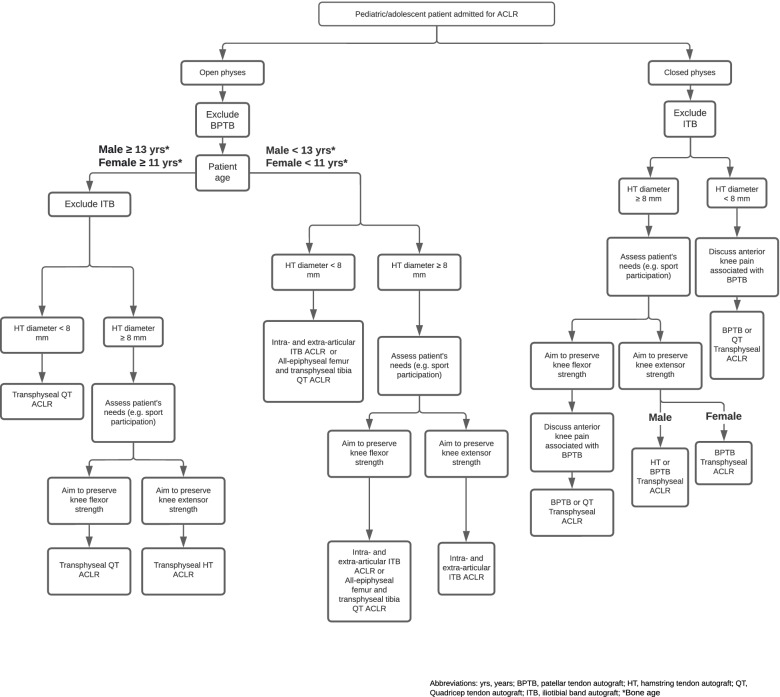
Simplified graft selection flowchart for ACLR in pediatric/adolescent patients. Pediatric and adolescent patients admitted for ACLR receive autografts best suited for their anatomical and personal needs. Physes status is first evaluated, followed by patient age. HT diameter, sex, sport participation and risk of anterior knee pain and surgical technique then guide final graft selection

Thinner autografts can lead to decreased graft incorporation throughout the healing process, increasing the likelihood for reinjury [20]. While graft diameter may be an issue for women compared to men, it has also been suggested to increase graft rupture rates in pediatric/adolescent patients compared to adults [22]. A systematic review and meta-analysis by Alomar et al. (2021) aimed to analyse the risk of ACLR failure secondary to graft rupture with hamstring autografts at cut-of graft diameters of 6, 7, 8, and 9 mm [23]. A total of 15 studies were included in the quantitative analyses of the meta-analysis. The results of the review showed significantly higher ACLR failure rates in patients receiving a HT with a graft diameter of < 7 mm compared to those receiving a HT with a graft diameter of ≥ 7 mm (*p* = 0.005) [23]. The results of this review also showed a significantly higher risk of ACLR failure in patients < 20 years old compared to patients ≥ 20 years old (*p* = 0.01) [23]. The authors of this review concluded that hamstring grafts used for ACLR should have a diameter of > 7 mm [23]. However, in patients aging < 20 years old or in patients participating in vigorous physical activity, surgeons should aim to use larger diameter grafts for ACLR [23]. While there is little evidence on what is the ideal graft diameter for pediatric ACLR, surgeons who factor in graft size in decision making prior to ACLR typically hold the threshold for graft diameter at 8 mm for pediatric and adult patients [22, 24]. Therefore, grafts with diameters measured at < 8 mm should lead surgeons to look for alternatives, as per our graft selection flowchart (Fig. 1). To do so, it is of interest to determine graft diameter preoperatively. This, however, has its own challenges, particularly with regards to measurement accuracy.

A cohort study by Partan et al. (2021) investigated the accuracy of MRI measurements and patient demographics in estimating semitendinosus and gracilis cross-sectional areas in patients (*n* = 51) under 18 years of age. The results of this study showed that the average of the three MRI measurements for each patient (at the level of the joint line, 3 cm cephalad to the medial tibial plateau, 5 cm cephalad to the medial tibial plateau) predicted intraoperative graft size within 0.5 mm in 72.5% of patients and within 1 mm in 96.1% of patients [22]. When adding age, sex, height and weight to MRI measurements, intraoperative graft size was predicted within 0.5 mm in 78.4% of patients and within 1 mm in 98% of patients [22]. The ability to accurately measure graft diameter preoperatively would allow surgeons to evaluate alternatives, such as the use of a different autograft or an allograft.

It is necessary to evaluate the patient’s physes status when determining ACL fixation technique. When using a soft tissue graft such as the HT or the QT autografts, large tunnel sizes or transphyseal hardware fixation can lead to growth disturbances [25]. Girls with a bone age of ≤ 10 years and boys with a bone age of ≤ 12 years are recommended to undergo physeal sparing procedures such as an all-epiphyseal ACLR [26]. An all-epiphyseal reconstruction should be performed when the diameter of the HT autograft used will be ≥ 8 mm, requiring a large tunnel size, or when the patient has significant growth remaining. This technique would help minimize the risk of physeal injury in skeletally immature patients [27]. When performing this technique, both tibial and femoral tunnels are drilled retrograde, entirely within the epiphysis [27]. The fixation of the graft is achieved with soft-tissue buttons on the femur and the tibia [27]. As for boys with a bone age of 15 or 16 years and girls with a bone age of 13 or 14 years, standard procedures are tolerated such that grafts with bone blocks such as the BPTB autograft can be used [26]. Transphyseal techniques are suggested for boys with a bone age of 13 or 14 years and girls with a bone age of 11 or 12 years [26].

### Clinical outcomes driving graft selection

Patient-related factors aside, surgeons may base their graft choice for ACLR on re-rupture rates, and RTS rates, including strength and functional outcomes recovery of different grafts studied in previous literature. The HT and BPTB autografts are the most popular grafts used in ACLR due to their ease of harvest, low failure rates, good physical and functional outcomes, and high rates of RTS [20, 28]. Despite there being many factors driving graft selection, there is no clear evidence explaining graft selection for ACLR, but patients and surgeons should discuss graft choice while considering the aforementioned factors to ensure a successful recovery and RTS. It would be of interest for surgeons to follow a pre-surgical graft selection framework (Fig. 1). As such, surgeons can avoid basing graft selection on their professional experiences and preferences. Instead, the patients’ characteristics and needs would be factored into the graft selection, which would hopefully lead to better postoperative outcomes such as early RTS and fewer graft ruptures. Many other factors such as the surgeon in charge of the operation and the surgical techniques used remain factors to be considered when evaluating the quality of postoperative outcomes. To the best of our knowledge, there are not many processes that have been developed for presurgical graft decision-making. At least one research group has used an individualized approach for graft selection. When planning for individualized ACLR, surgeons take into consideration the patient’s lifestyle, preferences, and activity level, as well as certain anatomical features (insertion site size and intercondylar notch dimensions), surgical techniques (single- or double-bundle ACLR) and graft choice [28–30]. The main goal of this practice is to restore the patient’s ACL to its native anatomy and function more precisely [28–30].

### Factors to consider for allograft vs autograft

The two main categories for ACLR graft choice are allografts and autografts. Ellis et al. (2012) compared the revision rates, function, activity, and satisfaction of BPTB autografts to BPTB allografts for ACLR in 79 patients 18 years or younger with closed physes. The allograft group was 15 times more likely to require reconstruction than the autograft group within one year follow-up (*P* = 0.001). However, 5 of the 7 allograft failures were due to premature RTS, before clearance from the senior surgeon [31]. One other allograft rupture was due to a patient performing high-intensity endurance exercises prematurely [31]. Two years postoperatively, survivorship for autografts was 96%, while it was 65% for allografts [31]. Four years postoperatively, this study shows evidence for equivalent function, activity level, and patient satisfaction when ACLR was successful with either allografts or autografts [31]. Given the delayed biological healing and incorporation of allografts (revascularization and recellularization), the authors attributed a delayed rehabilitation protocol and RTS to the allograft group [31]. As such, the delayed incorporation of allografts may contribute to their increased risk for re-rupture in athletes who return to a sport prematurely. Given the lack of adherence to postoperative guidelines and rehabilitation protocols and premature RTS observed in this study, it is difficult to conclude that there is more ACL re-injury in patients receiving allografts than in patients receiving autografts. In a cohort study conducted by Barrett et al. (2011), ACLR failure rates were compared between patients receiving allografts, BPTB autografts, and HT (semitendinosus/gracilis) autografts. The participant pool was separated in two categories: patients 25 years of age (*n* = 224) and under and patients over 25 years old (*n* = 195) [32]. For the purpose of this literature review, only the results pertaining to the category of patients 25 years and younger (average, 17.77; range 12–25) will be focused on. Within this patient group, allografts resulted in higher failure rates than BPTB autografts (*P* = 0.024) [32]. HT autografts also resulted in significantly higher failure rates than BPTB autografts (*P* = 0.036) [32]. Allografts and HT autografts failure rates were not significantly different [32]. These findings suggest that autografts are more appropriate than allografts for ACLR in younger patients. Additionally, the authors concluded that BPTB autografts are a preferable option for ACLR in patients 25 years and younger, given the higher failure rates observed in patients receiving HT autografts [32]. While many surgeons and investigators label graft failure rate as the most important factor in graft selection for ACLR, other factors are to be considered such as postoperative strength deficits, donor-site morbidity, and effects on growth in young patients with open physes [33].

A retrospective cohort study by Engelman et al. (2014) aimed to identify risk factors related to graft failure in 73 patients who underwent ACLR between the ages of 11 and 18 with having received allografts (*n* = 38) or autografts (*n* = 35). While there was a higher relative risk for failure to reach previous activity level (1.45; 95% CI, 0.63–3.32; *P* = 0.38) and reinjury in the allograft group (1.71; 95% CI, 0.77–3.79; *P* = 0.17), differences between groups were not statistically significant [34]. IKDC and Lysholm scores were similar between groups as well [34]. However, there were more graft failures in the allograft group (28.95%) than in the autograft group (11.43%). The allograft group was 4.4 times more at risk for revision surgery [34]. A similar risk was calculated by Cruz et al. (2020) who conducted a systematic review and meta-analysis including some of the aforementioned studies. When aiming to quantify the failure rates of allografts versus autografts in patients 19 years and younger having underwent ACLR with 2 years follow-up, Cruz et al. (2020) found a 3.87-fold increased risk of failure for allografts compared with BPTB or HT autografts [35]. Multiple hypotheses have been made to explain the increased failure rate in allografts for ACLR such as irradiation of chemical processing, but no clear conclusions can be drawn at this point due to a paucity of data [34, 36–38].

Based on their often-lower failure rates and shorter time to incorporate compared to allografts, autografts seem to be favored for ACLR in skeletally immature patients and any patient who will RTS or live an active lifestyle [31, 34, 38–40]. Autografts, however, have been reported to lead to short-term muscle deficits, donor site morbidity, and anterior knee pain [39, 40]. As for allografts, they tend to take more time to incorporate, but they also increase the risk for transmissible diseases, increased cost, and unfavorable tissue reaction [39, 40]. The lower or absence of donor-site morbidity in patients receiving allografts as well as the ease of availability of allografts make allografts a preferable option for older or relatively inactive patients, who will not RTS. Thus, allografts are not included in the graft selection flowchart designed for patients under 18 years of age (Fig. 1).

### Factors to consider for autograft selection

#### QT vs BPTB Autografts

Hunnicutt et al. (2019) compared QT autografts to BPTB autografts regarding patient recovery and functional outcomes in a group of 30 patients (19 male, 11 female) aging between 14 and 41 years old (median age, 22 years) after 6 to 23 months since surgery [41]. This cohort study demonstrated that there were no significant differences between groups for limb symmetry indexes (LSI) of the cross-sectional areas (CSA) of quadriceps muscles [41]. There were no differences between groups for central activation, with both groups achieving what is considered to be normal volitional activation of the quadriceps motor neuron pool (> 95%) [41]. Hop test performance, step length symmetry, and knee extensor strength using isokinetic dynamometry were found to be similar between both groups as well [41].

Han et al. (2008) conducted a similar study, investigating the differences between QT autografts and BPTB autografts on postoperative motion and anteroposterior knee stability, activity and functional scores, quadriceps muscle strength and incidence of anterior knee pain, and other complications. Both groups comprised 72 patients with ages ranging from 15 to 51 years (mean 27.8) [42]. The authors found no differences between groups in subjective satisfaction, anterior stability, function, or graft failure, as well as no differences in quadriceps strength recovery between groups with both groups recovering quadriceps strength to approximately 80% one year postoperatively as evaluated by peak torque ratio determined by Cybex isokinetic testing [42]. However, there was a lower incidence of anterior knee pain during various activities and kneeling pain within the QT group [42].

Based on these observations, both QT and BPTB autografts seem to be effective for ACLR. Evidently, more research needs to be conducted, specifically focusing on pediatric patients, to assess the short- and long-term effectiveness of QT and BPTB grafts in getting patients to RTS, preventing re-injury, recovering muscle strength, and achieving good patient satisfaction. The higher incidence of anterior knee pain in patients receiving the BPTB graft needs to be considered preoperatively for many reasons. In fact, patients suffering with anterior knee pain have also reported higher levels of anxiety, which have exacerbated the pain, decreased knee range of motion, limp, limb atrophy, knee swelling, and decreased quality of life [43, 44]. There are several suggested etiologies of the reported anterior knee pain in patients receiving BPTB autografts for ACLR, such as frequent bone-harvesting site pain, patellar tendinopathy, and neuroma due to a perioperative lesion to the infrapatellar branch of the medial saphenous nerve [45]. Given that other factors were found to be equal between QT and BPTB autografts in the aforementioned studies, anterior knee pain should be factored into the graft selection process [46]. Surgeons should discuss with their patients regarding how such morbidities can impact their quality of life prior to conducting ACLR and further evaluate other graft options and less invasive surgical techniques [46].

#### QT vs HT Autografts

Fischer et al. (2018) compared isokinetic quadriceps and hamstrings muscle strength in patients (*n* = 124) following ACLR who received either HT autografts (*n* = 63) or QT autografts (*n* = 61). The patients in the QT group were between 14 and 56 years old (21 ± 7.4), while the patients in the HT group were between 11 and 41 years old (21.5 ± 6.9), with no significant differences between groups and no effect of age [47]. Given the site of harvesting, it is expected that peak knee flexion torque would be lower in the HT group whereas peak knee extension torque would be lower in the QT group postoperatively. Peak isokinetic knee flexion torque of the injured knee at 60 deg/sec (Nm) was similar between groups at both timepoints [47]. The QT group had significantly lower values in peak torque knee extension of the injured knee within the first year after ACLR when compared to the HT group at 60 deg/sec (Nm) [47]. Consequently, the QT group had significantly higher hamstrings to quadriceps (H/Q) ratios (t1 = (82.4 ± 18.1)% & t2 = (72.3 ± 15.2)%) within the first-year post-ACLR than the HT group (t1 = (66.8 ± 15.7)% & t2 = (63.7 ± 12.4)%) [47]. A higher H/Q ratio in the QT group suggests better muscle balance around the knee joint and may be a protective factor in recurrent ACL injuries [47]. Both groups showed restored flexion torque to an adequate strength level with a side-to-side difference of less than 10% within one and two years post-ACLR [47]. In fact, LSI values in flexion were normal at both timepoints in the QT group, but only normalized at t2 (7.6 ± 1.6 months) in the HT group [47]. At t1 (5.5 ± 1.2 months) and t2 (7.6 ± 1.6 months), LSI values in extension were abnormal in both groups [47]. Given that both graft choices had a similar effect on peak knee flexion torque in both groups but that the QT group suffered significantly greater knee extension strength deficits than the HT group, patient-related factors such as sport preference should be factored in the graft selection process. If this is not a factor to consider for a patient in need of ACLR, the results of this study suggest that the advantage should be given to the QT autograft over the HT autograft given that the H/Q ratios were significantly higher in the QT group, but remain limited in generalizability due to a non-specific sample group.

Martin-Alguacil et al. (2018) aimed to compare the strength recovery and functional outcomes of HT and QT autografts using an anatomic single-bundle reconstruction for ACLR in 51 competitive soccer players. The patients in the QT group (*n *= 26) were 18.7 ± 3.6 years old, while the average age in the HT group (*n *= 25) was 19.2 ± 3.6 years [48]. The H/Q isokinetic ratios were significantly different between groups at all time points [48]. The QT group achieved the recommended ratios of ≥ 1.0 as early as 3 months postoperatively while the HT group achieved a ratio just below 1.0, even after 12 months postoperatively [48, 49]. While the HT group showed greater increases in peak torque in extension at all three measured velocities (60°/s, 180°/s, 300°/s) than the QT group at 3- and 6-months follow-up, there were no differences between groups at 12 months follow-up in peak knee extension torque [48]. Therefore, differences in quadriceps muscle strength between groups cannot account for the differences measured in H/Q ratios between groups at 12 months postoperatively. Differences between groups in changes of peak torque in flexor muscles from baseline to follow-up were not significantly different at any velocity or time point [48]. However, flexion strength deficits were observed solely in the HT group [48]. Perhaps a more favorable hamstring muscle strength recovery post ACLR in the QT group may explain the differences in H/Q ratios measured in this study [48]. The results of this study suggest that the flexion strength deficits in the HT group and thus the H/Q imbalance measured in this group increase the risk of graft rupture in patients similar to those included in this randomized control trial (young soccer players) [48].

#### HT vs BPTB Autografts

Salem et al. (2019) conducted a cohort study including 256 female patients aging between 15 and 25 years old. One hundred seventy-five women received BPTB autografts, while 81 women received HT autografts. In the BPTB group, 140 women were under 21 years old, while 63 women in the HT group were below 21 years of age [50]. Two important observations were made: The first is that while fifteen patients in the BPTB group (12%) reported extreme difficulty or an inability to kneel on the anterior side of the knee, this was the case for 1 patient (2%) in the HT group [50]; the second is that graft ruptures in the BPTB group were significantly lower than in the HT group below 21 years old, but not in patients aging between 21 and 25 [50].

Aune et al. (2001) compared HT autografts to BPTB autografts for ACLR in 72 patients. The HT group showed better isokinetic extension torque than the BPTB group at 6 months follow-up, but not at one or two years follow-up [17]. When measuring knee flexion torque at 60 deg/sec, expressed as LSI, the BPTB group only did better than the HT group at 6 months [17]. Similarly to the Salem et al. (2019) study, there was less donor-site kneeling pain reported in the HT group in comparison to the BPTB group after 2 years [17].

Hanada et al. (2019) measured differences in knee extension and flexion strength between groups by LSI (injured/non-injured) in favor of the HT group at 6 months follow-up, while there were no differences measured between groups for this parameter preoperatively and 1 year postoperatively [18]. The BPTB group’s lower flexion force may be a result of greater anterior knee pain in knee flexion position [18]. In fact, there is evidence suggesting that BPTB autografts lead to donor site morbidity such as knee pain when walking and kneeling, affecting knee function and patient quality of life [44]. Despite the wide age range included in this study, the authors included age in their analysis. Age seemed to be in association with the strength of knee extension at the final follow-up, but not at baseline, in favor of the younger participants [18]. There was no association drawn between age and knee flexion strength [18]. Taken together, the results of these studies all concord to conclude that BPTB autografts provide lower graft rupture rates but higher donor site morbidity than HT autografts.

Besides considering donor site morbidity and rupture rates in favoring HT graft over BPTB or vice-versa, skeletal maturity is another important factor to consider. Many surgeons have leaned towards the use of a HT graft to perform an ACLR in skeletally immature patients to avoid harvesting a patellar tendon from an open tibial tubercle apophysis and to avoid the bone plug or interference screw from crossing and potentially arresting the physis [51]. Additionally, a lack of understanding of growth remaining in patients with open physis influences surgeons to favour other grafts for ACLR, such as the HT autograft. However, this may put these patients at a higher risk for graft rupture because the hamstring tendons used are thinner. Therefore, the BPTB autograft is popular in ACLR for adolescent patients nearing skeletal maturity and returning to high-risk activities [51]. In patients with closed physes, the findings discussed are inconsistent. Two notable findings can help drive graft selection when deciding between HT and BPTB autografts for patients with closed physes. First, fewer graft ruptures in patients receiving BPTB autografts suggest that this graft may be preferred for patients returning to sport or in patients who will participate in moderate to vigorous physical activity [50]. Second, the donor-site morbidity and anterior knee pain often reported in patients receiving BPTB autografts suggest that the HT graft should be considered in patients who believe these morbidities would negatively affect their quality of life [17, 18]. As such, the surgeon should discuss the pros and cons of each graft with the patient preoperatively.

#### ITB vs BPTB vs HT vs QT Autografts

While BPTB autografts are not used for ACLR in patients with open growth plates, ITB reconstruction is tailored for patients with significant growth remaining as it is a physeal-sparing technique. The ITB autograft is usually limited to patients with roughly two years of growth left or, more objectively, open epiphyses. Typically, girls with a bone age of ≤ 10 years and boys with a bone age of ≤ 12 years are eligible to receive the ITB autograft [26]. A study from Sugimoto et al. (2019) investigated functional performance such as dynamic balance and function hop tests among patients < 22 years old who underwent ACLR with BPTB (*n* = 19), HT (*n *= 108), or ITB autografts (*n* = 33). The BPTB group demonstrated significant deficits in anterior reach performance in the dynamic balance test within limb and between groups [19]. Significant decreases in single-leg and cross-over hop tests in the operated limb compared to the uninvolved limb were measured in both the BPTB and HT groups [19]. All three groups demonstrated significant deficits in the triple hop test between limbs [19]. Given the results of the study, the authors suggest that the ITB autograft is comparable, if not more favorable, than the BPTB and HT autografts for ACLR in skeletally immature patients 6 to 9 months after surgery [19].

A follow-up study by Sugimoto et al. (2020) compared thigh circumference, knee range of motion (ROM), and quadriceps, hamstrings, hip abductor, and hip-extensor strength at 6 to 9 months postoperatively in patients under 22 years old who were given BPTB (*n* = 20), HT (*n* = 111), or ITB (*n* = 33) autografts for ACLR. The BPTB group demonstrated significant deficits in quadriceps strength compared to both other groups, while the HT group demonstrated significant deficits in hamstrings strength compared to the two other groups [52]. As such, strength deficits measured in this study were directly related to the location of the graft harvested [52]. While more research is needed comparing the ITB autograft to other autografts in pediatric patients with skeletal maturity equivalent age groups, both Sugimoto's studies concur to suggest that the current evidence is in favor of the ITB autograft for this patient population [19, 52]

A recent retrospective study by Wren et al. (2021) evaluated knee extensor function post-ACLR in pediatric patients (*n* = 145) receiving ITB, BPTB, HT or QT autografts. Patients included in the studied were aging between 7 and 21 years old [53]. As hypothesized by the authors, knee extensor function recovery was significantly greater in patients receiving autografts that do not disrupt the knee extensor mechanism, being the ITB and HT autografts [53]. Patients receiving an ITB autograft demonstrated higher knee flexion angles on the operated vs contralateral side during drop jump and cutting than all other patient groups [53]. Decreases in loading on the injured limb were noted in all four groups, but not evenly. The ITB group showed the slightest decrease, followed by the HT group, the QT group and finally, the BPTB group [53]. No between-group differences were observed regarding loading on the contralateral limb, which increased similarly in all four groups [53]. The ITB group also showed significantly lower kinetic asymmetry at the lower limbs compared to both the QT and BPTB groups (*p* ≤ 0.005) [53]. The HT group showed similar results to the ITB group [53]. Only knee extensor moments differed significantly between the ITB and the HT groups (*p* ≤ 0.05) while no asymmetry was objectified between the QT and BPTB groups (*p* ≥ 0.16) [53]. The authors did include sub-analysis for patients receiving isolated ACLR, meniscus repair, or in patients younger than 16 years of age. The most important finding of the study was that patients receiving an ITB autograft exhibited the fastest knee extensor recovery, with the HT group coming in second. Despite all these interesting findings, knee extensor recovery is not the only factor that would drive surgeons to favor one graft over another. The authors were not able to include any information on failure rates between groups. A follow-up study would most definitely provide important information driving ACLR graft selection in this patient population.

#### Surgical technique

In order to avoid the high failure rates observed in pediatric patients and to optimize recovery post-ACLR, Kocher et al. (2018) described a physeal-sparing surgical technique that consists of intra- and extra-articular ACLR using the ITB autograft in young patients. A retrospective study by the same authors evaluated the effectiveness of this surgical technique in 237 patients ageing between 5.7 to 15.6 years of age [54]. The mean Pedi-IKDC scores were 93.3 ± 11.0 and the mean Lysholm instrument scores were 93.4 ± 9.9 [54]. A 96.5% of patients returned to sport [54]. Out of 240 knees included in the analysis, graft rupture information was collected for 137 knees within 6 years follow-up [54]. Only 9 ruptures were observed, with 8 of the 9 ruptures being due to contact during sport and 1 being due to having stepped awkwardly off a curb [54]. The 6.6% rupture rates measured by Kocher et al. (2018) compared to the higher failure rates of all-epiphyseal techniques, which typically range between 11 to 17%, suggest that this physeal-sparing technique be favoured in prepubescent children with open growth plates (Fig. 1) [54]. The technique described by Kocher et al. (2018) also avoids growth arrest, physeal damage, and angular deformities that have been observed in prepubescent patients after all-epiphyseal, partial epiphyseal or transphyseal ACLR [54]. Transphyseal techniques have therefore been regarded as the standard ACLR surgical technique for patients with little to no growth remaining regardless of the graft chosen (Fig. 1) [55–57]. As for patients with significant growth remaining, a hybrid surgical technique comprising of an all-epiphyseal femoral tunnel combined with a traditional transphyseal tibial tunnel can be performed to help reduce the risk of growth arrest (Fig. 1) [58].

### Considerations for pre/rehabilitation protocols

Many studies included in this review have observed postoperative strength deficits related to the location of the graft harvested. Rehabilitation protocols should be adapted to patients based on the graft they received to restore any strength deficit observed postoperatively. The patient’s knee strength should not only come to mind postoperatively. Graft selection should consider the patients’ needs, such as which sport they participate in, to avoid weakening muscles necessary for that specific activity. Measuring knee flexion and extension torque preoperatively and prescribing a prehabilitation protocol can also help minimize postoperative strength deficits and favoring a healthy H/Q ratio to prevent reinjury [59].

## Conclusion

The main finding of this review is that there is a lack of evidence surrounding the comparisons of different autografts on functional outcomes, failure rates and patient satisfaction for pediatric ACLR. Despite that there is still no consensus regarding the best graft for ACLR, the findings discussed in this review suggest that the ITB autograft deserves to be considered for ACLR in skeletally immature patients while the BPTB graft would be reserved for patients with little to no growth remaining. The QT, HT, and BPTB autografts lead to specific short-term muscle strength deficits post-ACLR that should be considered concomitantly with patient specific factors. Adequate rehabilitation protocols can help minimize those muscle strength deficits. There is a consistent reporting of anterior knee pain or kneeling pain in patients receiving a BPTB autograft, but the latter has led to a lower re-rupture rate than its counterparts, which explains its widespread use for ACLR. The lack of research evaluating graft comparisons for ACLR in pediatric patients along with inconsistent results across different studies does not allow us to conclude definitively what is the best approach for this specific procedure and age group. Additionally, as many of the articles included in this review include samples of patients from various age groups, the findings are not generalizable to the pediatric population. Moving forward, as age and sex have been identified as factors influencing ACLR recovery and RTS, studies should narrow their population group to specific age groups rather than include pediatric patients and adults within the same pool and they should consider male and female patients separately. It is imperative to conduct studies with consistent fixation and surgical techniques and rehabilitation protocols to draw reliable comparisons between the grafts. While more evidence is needed regarding ACLR in skeletally immature patients, individualized graft selection for pediatric ACL-R may offer a suitable approach for patients to achieve optimal outcomes.

## Abbreviations

ACLR: Anterior cruciate ligament reconstruction
ACL: Anterior cruciate ligament
QT: Quadriceps tendon
HT: Hamstrings tendon
BPTB: Patellar tendon
ITB: Iliotibial band
SF-36: Short Form-36
SF-36 BP: Short Form-36 bodily pain
RTS: Return to sport
LSI: Limb symmetry index
CSA: Cross-sectional area
H/Q: Hamstrings to quadriceps
